# Why blood flow restriction cuff features are an important methodological consideration- a short commentary on “cerebral cortex activation and functional connectivity during low-load resistance training with blood flow restriction: an fNIRS study”

**DOI:** 10.3389/fphys.2024.1482816

**Published:** 2024-12-04

**Authors:** Nicholas Rolnick, Matthew Clarkson, Luke Hughes, Vasileios Korakakis, Victor De Queiros, Stephen D. Patterson, Samuel Buckner, Tim Werner, Dahan Da Cunha Nascimento, Sten Stray-Gundersen, Okan Kamiş, Mathias Thoelen, Kyle Kimbrell, Ewoud Jacobs

**Affiliations:** ^1^ The BFR Pros, New York, NY, United States; ^2^ Department of Exercise Science and Recreation, CUNY Lehman College, Bronx, NY, United States; ^3^ Institute for Health and Sport, Victoria University, Melbourne, VIC, Australia; ^4^ Department of Sport, Exercise and Rehabilitation, Northumbria University, Newcastle upon Tyne, United Kingdom; ^5^ Department of Health Sciences, PhD in Physiotherapy Program, University of Nicosia, Nicosia, Cyprus; ^6^ Graduate Program in Health Sciences, Federal University of Rio Grande do Norte (UFRN), Natal, Brazil; ^7^ Centre for Applied Performance Sciences, St. Mary’s University Twickenham, London, United Kingdom; ^8^ USF Muscle Laboratory, Exercise Science Program, University of South Florida, Tampa, FL, United States; ^9^ Department of Exercise Science, Salisbury University, Salisbury, MD, United States; ^10^ Department of Physical Education, Catholic University of Brasilia (UCB), Brasilia, Brazil; ^11^ Department of Exercise Science, University of South Carolina, Columbia, SC, United States; ^12^ Department of Sports and Health, Aksaray University, Aksaray, Türkiye; ^13^ Department of Physical Therapy, Anna TopSupport, Eindhoven, Netherlands; ^14^ Owens Recovery Science, San Antonio, TX, United States; ^15^ Department of Rehabilitation Sciences, Ghent University Faculty of Medicine and Health Sciences, Ghent, Belgium

**Keywords:** BFR training, multi-chambered design, commentary, arterial occlusion pressure, limb occlusion pressure

## Introduction

We read with great interest the recent study titled “Cerebral cortex activation and functional connectivity during low-load resistance training with blood flow restriction: An fNIRS study” published in PLOS ONE earlier this year (Jia et al., 2024). The study adds to our limited understanding of the cerebral demands of blood flow restriction (BFR) exercise and the potential role of applied pressure. The authors examined cerebral oxygenation levels following squat exercise performed at 30% of one repetition maximum, with bilateral BFR applied at 150, 250, and 350 mmHg using the B-Strong cuffs (B-Strong, USA). The authors noted enhanced cerebral oxygenation levels in many cortical regions which dropped sharply when 350 mmHg was applied. In addition, they also found the existence of an interaction effect of pressure on cortical activation in the primary motor cortex, pre-motor cortex, and supplementary motor cortex whereas there was a less pronounced effect in the dorsolateral prefrontal cortex. The authors should be commended for their pioneering investigation into the relationship between applied BFR pressures and cortical demands. However, we wish to bring up some methodological concerns and considerations regarding the cuff utilized as well as the way that pressure was applied in data collection and speculate on its potential impact and influence on the ultimate outcomes as calculated and reported in this study.

In the last decade, BFR has grown in popularity in multiple practice settings (Scott et al., 2023). As a result of this popularity, BFR cuff manufacturers have begun to produce different types of BFR equipment and incorporate device features that can impact the acute and/or longitudinal responses to BFR exercise (Rolnick et al., 2023). Features such as autoregulation of applied BFR pressures during exercise (Hughes et al., 2024; Jacobs et al., 2023), cuff material and width (Buckner et al., 2017; Loenneke et al., 2012) or changes in the bladder design that houses the air that is applied to the limb (Dancy et al., 2023) have received increased attention.

Jia et al. (2024) utilized the B-Strong cuff, a multi-chambered BFR cuff that is designed to avoid significant arterial occlusion to promote user safety during its application (Rolnick and Cerqueira, 2021). These are distinct from single air bladder (e.g., a traditional tourniquet) cuffs that are designed to determine a personalized pressure (Limb occlusion pressure, LOP) during BFR exercise (Patterson et al., 2019). LOP has been defined as the minimum applied pressure needed to fully occlude arterial and venous blood flow to an extremity, and provides a way to standardize BFR application (Patterson et al., 2019). Personalizing the pressure application has been recommended in clinical practice and research because it allows for similar comparisons between participants and can assist practitioners in implementing applied pressures that influence relevant physiological outcomes. LOP values are largely predicated on the BFR cuff width and each participant’s resting blood pressure, limb circumference, and body position (Graham et al., 1993; Hughes et al., 2018; Loenneke et al., 2013; Sieljacks et al., 2018). Relativizing the applied pressure for each individual using the LOP approach ensures these participant characteristics are taken into consideration and can provide a better estimation of the applied pressure and the extrapolation and comparison of findings between conditions and laboratories. While the absolute amount of pressure applied to each participant may vary significantly when standardizing the pressure application to a percentage of LOP between cuffs of different sizes, the physiological stimulus appears similar (Loenneke et al., 2012). For example, 250 mmHg applied pressure to one individual may be complete occlusion whereas it may only be partial occlusion to another individual based upon individual characteristics. Therefore, an important methodological consideration when looking to investigate the impact of pressure on a variety of physiological responses, including cerebral oxygenation, is utilizing cuffs and methods that can relativize the applied BFR pressure.

As the primary goal of the current study was to determine the pressure-dependent relationship to cortical activation and cerebral oxygenation, the use of a multi-chambered cuff without a standardized method to relativize the applied pressure could impact any potential effect observed from increasing pressure compared to a single-chambered bladder BFR cuff. Prior research has shown arterial blood flow only begins to be modified from resting conditions with greater than 350 mmHg of applied pressure when using multi-chambered BFR cuffs (Citherlet et al., 2022). Conversely, pressures as low as ∼86 mmHg (40% LOP in this particular study) were shown to modulate blood flow from resting conditions in the Hokanson device (Citherlet et al., 2022). It is tempting to suggest that 350 mmHg with a multi-chambered bladder BFR cuff and 40% LOP with a single-chambered bladder BFR cuff provide a similar physiologic stimulus. However, without instituting methods to relativize the applied pressures in the multi-chambered cuff, it is difficult to know. Nonetheless, if this comparison is true, it would suggest that a pressure of 350 mmHg in a multi-chambered BFR cuff, which is on the low end of the recommendations for applied pressure during BFR exercise using single-chambered cuffs (40%–80% LOP) (Patterson et al., 2019), alters cortical activation and cerebral oxygenation. Given the standard application of a fixed pressure *in lieu* of a relativized application, participants in Jia et al. (2024) were likely exercising at different levels of pressure relative to their LOP, creating uncertainty around the findings and its translation to practice.

We recommend that future studies either consider personalizing to a %LOP or standardize the cuff fitting pressure when using multi-chambered cuffs and attempting to elucidate pressure-dependent changes in outcome measures. At the very least, individual features, such as the participant’s resting blood pressure and limb circumference should be reported to provide greater context. Some research shows that multi-chambered cuffs can be personalized (Machek et al., 2022), so use of this cuff design feature in research studies to explore the role of applied pressure is not necessarily a methodological flaw but does require additional steps to contextualize (e.g., measurement of LOP). Conversely, applying an arbitrary amount of pressure for each condition reduces generalizability and limits the strength of the findings. This is particularly important considering the only pressure condition capable of reducing cerebral oxygenation and activity in the Jia et al. (2024) study represents not only the minimum pressure threshold needed to decrease resting arterial blood flow with the B-Strong cuffs (Citherlet et al., 2022), but also was the maximum pressure examined. Further, the authors did not mention this arbitrary pressure application approach as a limitation. The authors mentioned the method of compression and the material of the compression band, but we assert that the multi-chambered bladder design is the biggest limitation to this line of BFR research on pressure-dependent relationships. In summary, we commend the authors for investigating a novel component of BFR training but hope that highlighting the potential impact of cuff design and BFR methodology can have on the BFR stimulus spurs careful consideration of these factors and generates more standardization of BFR application in research settings.
